# Grinspan's Syndrome: A Rare Case with Malignant Transformation

**DOI:** 10.1155/2018/9427650

**Published:** 2018-03-04

**Authors:** Numan Kökten, Lokman Uzun, Ayşe Serap Karadağ, Tülay Zenginkinet, M. Tayyar Kalcıoğlu

**Affiliations:** ^1^Department of Otolaryngology, Istanbul Medeniyet University Medical Faculty, Goztepe Training and Research Hospital, Istanbul, Turkey; ^2^Department of Dermatology, Istanbul Medeniyet University Medical Faculty, Goztepe Training and Research Hospital, Istanbul, Turkey; ^3^Department of Pathology, Istanbul Medeniyet University Medical Faculty, Goztepe Training and Research Hospital, Istanbul, Turkey

## Abstract

**Aim:**

Oral lichen planus (OLP) is one of the common chronic inflammatory, noninfectious, and precancerous oral mucosal diseases that affect the stratified squamous epithelium in adults. Grinspan et al. reported an association of OLP with diabetes mellitus and vascular hypertension and called that Grinspan's syndrome in 1966. We aim to present a case of Grinspan's syndrome with malignant transformation.

**Case Presentation:**

A 60-year-old man who presented with a ten-year history of OLP diagnosed clinically and histologically was referred to our otolaryngology department with a painless swallowing in the left buccal mucosa for 3 months. Clinical examination revealed several plaques, striated white lesions in the buccal mucosa bilaterally, and an exophytic tumor in the left buccal mucosa. Histopathological examination showed lichen planus bilaterally and oral squamous cell carcinoma in the left buccal mucosa. The tumor had been developed on the preexisting areas of lichen planus which had been histologically proven before. The tumor was removed completely, and the tissue defect on the buccal mucosa was repaired with a split-thickness skin graft.

**Conclusion:**

Patients with OLP should be followed up periodically in a long term at close intervals for early diagnosis of malignant transformation.

## 1. Introduction

Oral lichen planus (OLP) is one of the common chronic inflammatory, noninfectious, and precancerous oral mucosal diseases that affect the stratified squamous epithelium in adults [1, 2]. In general population, OLP prevalence is about 0.5–2% worldwide and more common in females [2]. OLP can present as painless white streaks, raised, lacy-like lesions, or painful and persistent ulcers or plaques or papules and can resemble leukoplakia [2, 3]. In OLP patients, buccal mucosa, gingiva, tongue, labial mucosa, and vermilion of the lower lip are the most common affected sites [1–4]. The buccal mucosa is affected most commonly, and the tongue and gingiva follow buccal mucosa [4]. The lesions are mostly bilateral [5].

Although the etiopathogenesis is still unknown, immunological mechanisms are blamed [1, 6]. Autocytotoxic T lymphocytes were arraigned to trigger apoptosis of epithelial cells resulting in chronic inflammation. Civatte bodies, acanthosis, and parakeratosis are the results of lymphocytic infiltration of subepithelial tissue, basal membrane impairment, and degenerations of keratinocytes [1]. Malignancy potential of OLP is reported between 0.3 and 10% [7, 8]. The release of inflammatory cytokines caused by oxidative stress is supposed to activate transcription factors that affect premalignant cells to turn into malignant cells [1, 3, 4].

OLP diagnosis can be made with clinical features like classic oral white lesions, but to confirm diagnosis and exclude the dysplasia or malignancy, the histopathological examination is recommended [5, 6].

OLP has been associated with different systemic diseases such as primary biliary cirrhosis, thymoma, ulcerative colitis, chronic active hepatitis, and myasthenia gravis and with several viruses such as hepatitis C virus (HCV), human papillomavirus (HPV), human herpes virus 6 (HHV-6), and Epstein-Barr virus (EBV) [6]. The lesions related with an identifiable aetiology which resemble OLP clinically and histopathologically are called lichenoid reaction [9]. Some dental materials, chronic graft versus host disease, and drugs such as angiotensin-converting enzyme inhibitors, antimalarial drugs, and nonsteroidal anti-inflammatory drugs are some examples that cause lichenoid reactions [6, 9].

Anti-inflammatory agents such as the topical corticosteroids are mainly preferred for OLP treatment; if lesions are widespread, systemic agents may be required [1, 5, 6].

Patients with OLP should be followed up periodically in a long term at close intervals for early diagnosis of malignant transformation. Malignant transformation after 40 years from the initial diagnosis of OLP has been reported [6, 10].

Grinspan et al. reported an association of OLP with diabetes mellitus (DM) and vascular hypertension (VHT) and called that Grinspan's syndrome [11]. We present a case of Grinspan's syndrome with malignant transformation.

## 2. Case Presentation

**Figure 1:**
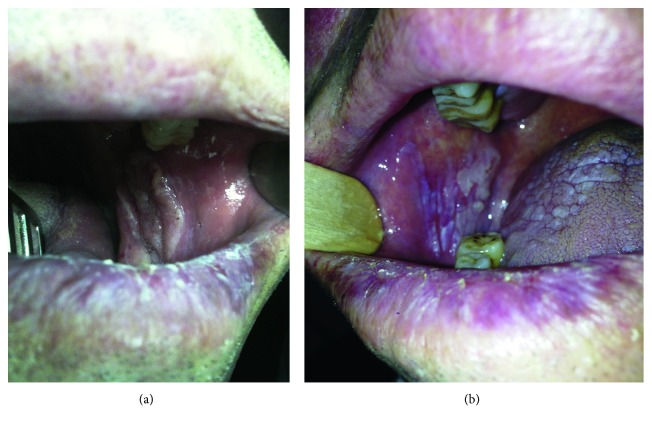
(a) Exophytic tumor with a smooth whitish surface on the left buccal mucosa. (b) Lichen planus in the right buccal mucosa and on the tongue; actinic cheilitis is also seen on the lips.

A 60-year-old man who presented with a 10-year history of OLP diagnosed clinically and histopathologically was referred to our otolaryngology department from dermatology department. The complaints of the patient were burning sensation after hot or spicy foods in the left buccal mucosa and painless swallowing in the left buccal mucosa for 3 months (Figure 1(a)). There was no history of using tobacco, drinking alcohol, or any other harmful habits. Medical history of the patient represents diabetes mellitus of 5 years and newly diagnosed hypertension accompanying OLP.

**Figure 2:**
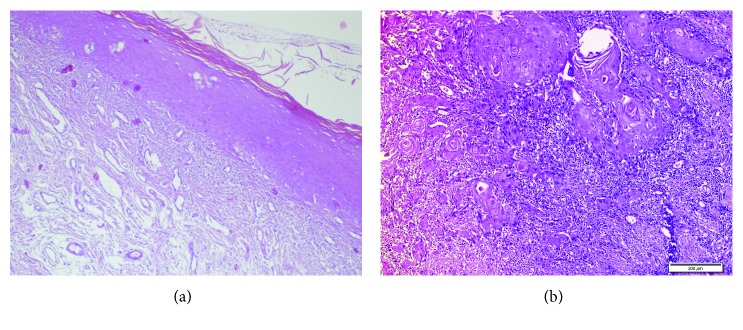
(a) H&E ×100: lichen planus with hyperkeratosis, parakeratosis, epidermal hyperplasia, eosinophilic colloid bodies (Civatte), and lymphocytic infiltration at superficial dermis. (b) H&E ×10: areas infiltrated with OSCC.

Clinical examination revealed several plaques and striated white lesions on the tongue and in the left and right buccal mucosae (Figure 1(b)) and an exophytic tumor with a smooth whitish surface on the left buccal mucosa. There was also actinic cheilitis on his lips. A complete otolaryngologic examination was done, and a blood sample was sent to laboratory for hematological examination. Incisional biopsies were taken from each buccal mucosa. Histopathological examination of the lesions showed lichen planus in the right and left buccal mucosae (Figure 2(a)) and oral squamous cell carcinoma (OSCC) in the left buccal mucosa (Figure 2(b)).

**Figure 3:**
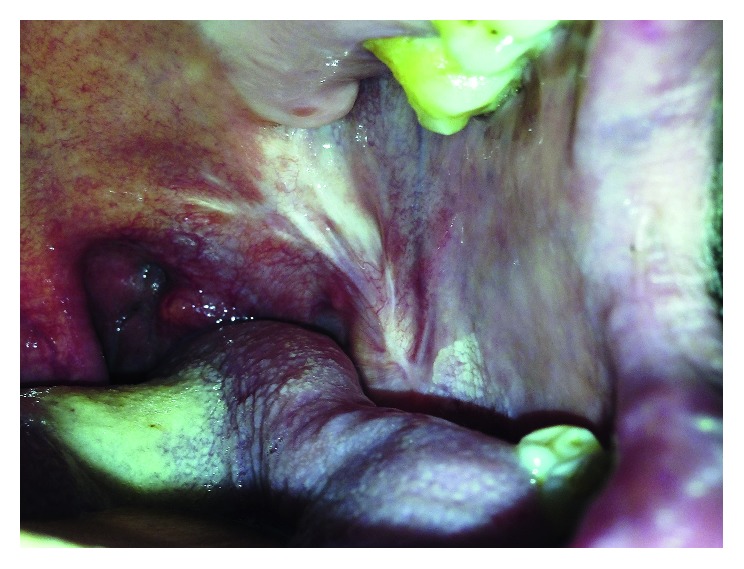
Left buccal mucosa one year after the operation. A scar tissue is present, and there is no sign for relapse.

The tumor had been developed on the preexisting areas of lichen planus which had been histologically proven before. The tumor was removed completely, and the tissue defect on the buccal mucosa was repaired with a split-thickness skin graft.  Figure 3 represents the situation of the left buccal mucosa one year after the operation.

## 3. Discussion

OLP may clinically demonstrate various forms such as reticular, erosive and ulcerated, atrophic, hypertrophic, bullous, and pigmentous forms [5, 6]. Reticular form is the most frequent form with small white papules or white lines network which is known as Wickham's striae [6]. Erosive and atrophic forms are less common but often associated with malignancy development [2, 4, 6].

The incidence of oral mucosa malignancies is reported about 0.004% per year with an 80% rate of OSCC in Europe [2]. OSCC may present like nonhealing, indurated ulcers or hyperkeratotic, exophytic masses or as submucosal, slightly indurated, red lesions with an intact epithelium less frequently [12]. Red areas include more frequent dysplastic features than white areas and should be biopsied preferentially [13]. Known external risk factors are poor nutrition, alcohol abuse, tobacco exposure, erythroplakia, and leukoplakia [12].

Nowadays, the malignant transformation of OLP is still a controversial subject [2, 5, 8]. The atrophic and erosive forms have more risks for malignant transformation [2, 4, 6]. In these forms of OLP, carcinogenic agents are supposed to react more easily because of the atrophy or absence of the epithelium [6]. Degeneration-healing cycles of cells in the OLP region are also assumed to facilitate neoplasm development.

Both OLP and lichenoid reactions can cause OSCC. In a recent meta-analysis of 57 studies with the data on 19,676 OLP and 419 oral lichenoid reaction cases, Aghbari et al. [14] reported that the rate of malignant transformation for OLP was 1.1% and 2.5% for the oral lichenoid reactions. They also reported that smokers, alcoholics, and HCV-infected patients have a higher incidence of malignant transformation.

Krutchkoff et al. had established the criteria of malignant transformation of OLP as follows: there must be a 2-year follow-up period after the initial clinical and histological diagnosis of OLP and the absence of carcinogenic factors such as exposure to tobacco and alcohol history [15]. Our patient had been followed up for OLP for 10 years and had not any external carcinogenic factor.

Diabetes mellitus (DM) is a clinical entity with hyperglycemia due to insulin defect. Disregulation of insulin, glucose, and lipids can lead to skin lesions in some DM patients [16]. Grinspan et al. reported an association of OLP with DM and VHT and called that Grinspan's syndrome [11]. However, with the presentation of three cases, drug therapy for VHT and DM was blamed to produce lichenoid reactions for Grinspan's syndrome [17]. In our case, OLP is associated with DM, VHT, and OSCC. Our patient had been followed up for OLP for 10 years and for DM for 5 years being treated with saxagliptinum 5 mg/day for 3 years and insulin 25 units/day at nights for 5 years. VHT is diagnosed just before the surgery and treated with perindoprilat-indapamide (5–12.5 mg/day). So, he did not use any DM or VHT drugs before the OLP appeared. Blood glucose levels of our patient are under control about 94–142 mg/dL at near-normal preprandial levels, and hemoglobin A1C levels are under 7% with the treatment. Systolic and diastolic blood pressures of our patient are under control with the treatment with a range of 122–166 mm·Hg and 78–115 mm·Hg, respectively. He continues the same treatment.

In our case, the patient was followed up for lichen planus earlier by dermatologists; after recognition of mass in the left buccal mucosa, he was referred to us. Hematological and histopathological investigations showed that the patient has Grinspan's syndrome with malignant transformation. The patient underwent surgical treatment and followed up monthly for the first year after the operation. Now, no relapse or any sign of metastasis was observed in the patient 33 months after the operation.

## 4. Conclusion

Malignant transformation is the most important complication of long-standing and nonhealing lichen planus. Close follow-up and repeat biopsies are mandatory for prevention and early recognition of malignant transformation. It is still controversial about Grinspan's syndrome that DM and VHT are accompanying OLP or medication of DM and VHT leads to oral lichenoid lesions. We presented a case with Grinspan's syndrome which appeared before DM and VHT medication and with malignant transformation.
